# Patellar Instability Following Total Knee Arthroplasty: A Review of Etiology, Diagnosis, and Management Strategies

**DOI:** 10.7759/cureus.89213

**Published:** 2025-08-01

**Authors:** Zubair Younis, Muhammad A Hamid, Balu Ravi, Raaghav Verma, Thomas Devasia

**Affiliations:** 1 Trauma and Orthopaedics, The Royal Wolverhampton NHS Trust, Wolverhampton, GBR; 2 Orthopaedic Surgery, University Hospitals Birmingham NHS Foundation Trust, Birmingham, GBR

**Keywords:** computer assisted surgery, femoral rotation, lateral retinacular release, patellar maltracking, total knee arthroplasty

## Abstract

Patellar instability remains one of the most challenging and persistent complications following total knee arthroplasty (TKA), significantly affecting patient satisfaction and functional outcomes. Despite advances in implant technology and surgical techniques, patellofemoral maltracking remains a leading cause of anterior knee pain and one of the most frequent non-infectious reasons for revision surgery. The etiology is complex and often involves a combination of component malposition, soft-tissue imbalance, patient-specific anatomical risk factors, and suboptimal implant design. Internal rotation of the femoral or tibial components is consistently associated with increased lateral patellar tilt and subluxation, while soft-tissue contributors such as lateral retinacular tightness or medial instability can exacerbate the condition. Diagnostic evaluation relies on a thorough clinical examination supported by imaging modalities such as CT, which is the gold standard for assessing component rotation. Intraoperative computer-assisted navigation offers real-time feedback and is increasingly being used to reduce malalignment-related complications. Management strategies vary according to the underlying cause, ranging from conservative physiotherapy in mild cases to component revision, soft-tissue realignment, and distal realignment osteotomies in more severe or complex scenarios. Outcomes are most favorable when surgical intervention is tailored to the specific etiology of instability. Preventative strategies, including precise rotational alignment, appropriate implant selection, and intraoperative tracking assessment, are crucial for optimizing patellar tracking and improving long-term TKA outcomes. This narrative review provides an integrated analysis of the biomechanical, diagnostic, and therapeutic considerations essential to managing patellar instability in contemporary TKA practice.

## Introduction and background

Total knee arthroplasty (TKA) is a surgical procedure performed to relieve pain and restore function in patients with advanced knee arthritis. However, postoperative complications can significantly affect outcomes, with patellar instability being one of the most challenging and frequently reported issues. Patellar instability refers to the abnormal movement of the patella relative to the femur, which may manifest as maltracking, subluxation, or dislocation. Proper patellar tracking is essential for ensuring smooth knee flexion and extension, and disruptions to this can result in pain, functional limitations, and implant failure.

Patellar instability remains one of the most challenging complications following TKA, with an incidence reported between 1% and 20% [1]. It significantly impacts postoperative outcomes and patient satisfaction. Despite advancements in surgical technique and implant design, patellofemoral instability continues to be the most commonly reported cause of anterior knee pain and remains the predominant non-infectious reason for revision TKA, accounting for as much as 35% of complications in earlier studies and persisting at rates of 1%-12% in more contemporary series [2].

Patella maltracking refers to the abnormal displacement of the patella from its central alignment, often presenting as excessive tilt, subluxation, or complete dislocation [3]. Biomechanical factors contributing to maltracking include imbalance between the vastus medialis obliquus (VMO) and vastus lateralis muscles, tightness of the lateral retinaculum, increased Q angle, trochlear dysplasia, patella alta, and rotational deformities of the femur or tibia. These factors disrupt the normal patellofemoral joint mechanics, leading to lateral deviation of the patella during knee flexion and extension.

Following TKA, maltracking can lead to anterior knee pain, particularly during activities such as stair climbing or rising from a chair, as well as increased prosthetic component wear, a heightened risk of loosening, patellar fractures, and joint instability [4,5]. Despite advancements in implant design and surgical techniques, complications involving the extensor mechanism remain prevalent and account for up to 50% of non-infectious revision procedures [2]. Crucially, extensor mechanism imbalance and maltracking are the most common causes of pain in TKA patients, frequently arising from component malposition or altered soft-tissue tensioning during surgery [2].

Recent in vivo tracking work has shown that elevating the tibial joint line by as little as 4 mm creates a pseudo‑patella baja, a condition where the patella appears lower due to joint line elevation rather than actual shortening of the patellar tendon. This shifts patellar contact distally and alters proximodistal and anteroposterior kinematics throughout flexion [5]. Computer‑assisted surgery (CAS) can display the native patellar path intraoperatively, allowing the surgeon to adjust bone cuts before final implantation and potentially avert postoperative maltracking [5].

This narrative review synthesizes current evidence on the multifactorial etiology of patellar instability after TKA. It also explores evidence-based diagnostic strategies and contemporary management algorithms, integrating biomechanical insights and outcomes from key clinical studies.

## Review

Etiology and risk factors

Component Malpositioning

Femoral component malrotation is the most consistent cause of patellar maltracking. Internal rotation displaces the trochlear groove medially, reducing patellar engagement during early flexion. Cadaveric models have demonstrated that as little as 3° of internal femoral rotation increases the lateral patellar tilt by 40% [6]. Berger et al. determined that an internal rotation of 3°-8° correlates with subluxation, while >7° leads to dislocation or early failure [7]. The epicondylar axis remains the most reliable landmark for femoral component rotation, particularly in valgus knees where lateral condylar deficiency renders the posterior condylar axis unreliable [8]. According to Eisenhuth et al., rotational alignment errors of the femoral component not only increase lateral retinacular tension but also disrupt the flexion axis of the knee, directly worsening extensor mechanism mechanics and increasing patellofemoral shear [2].

Tibial component malrotation also plays a crucial role. Internal tibial rotation lateralizes the tibial tubercle, exaggerating the Q angle and leading to lateral patellar tracking. This is often due to inadequate exposure of the posterolateral plateau or using the posterior tibial border rather than the medial third of the tibial tubercle as a landmark [2]. As Ferri et al. emphasize, residual valgus alignment and tibial internal rotation are among the most prevalent intraoperative technical errors leading to maltracking, especially in the absence of pre-existing deformity [1]. Combined femoral and tibial malrotation amplifies patellar instability, with internal rotation >10° associated with a 12-fold increase in revision rates [7].

Errors during patellar resurfacing can also contribute to instability. Lateralized placement increases lateral retinacular tension, leading to tilt or dislocation [9]. Asymmetric resection, particularly under-resection of the lateral facet, disrupts native biomechanics. The normal medial to lateral facet ratio is approximately 14:23 mm, and deviation induces tilt [10]. Overstuffing the patellofemoral joint, referring to an increase in the overall thickness of the patella and trochlear construct beyond its native dimensions, elevates shear forces by up to 25% when the patellar thickness increases by more than 2 mm [11]. Inappropriately thick patellar components increase lateral shift and contact stress, especially in the presence of a shallow trochlea, and should be avoided when resurfacing is performed [2].

Soft-Tissue Imbalance

Soft-tissue contributors include lateral retinacular tightness, commonly seen in chronic dislocations. Medial retinacular insufficiency, which may result from hematoma formation or aggressive postoperative physiotherapy, is another factor [4]. Quadriceps weakness, particularly atrophy of the vastus medialis obliquus, impairs dynamic medial stabilization [1]. Disruption of the medial patellofemoral ligament (MPFL) during medial parapatellar arthrotomy or closure can unbalance medial support and result in dynamic lateralization, especially in early postoperative motion [1].

Implant Design Considerations

Implant-related factors affecting patellar tracking include shallow trochlear grooves, which fail to contain the patella beyond 45° of flexion [12]. Symmetrical femoral components require extremely precise rotational alignment [13]. Mobile-bearing prostheses, though once theorized to improve tracking, have shown no consistent benefit over fixed-bearing designs [14]. Data from a systematic review confirm that symmetrical, shallow trochlear grooves are consistently associated with abnormal patellar kinematics and higher maltracking rates [1].

Patient-Specific Factors

Several intrinsic patient factors increase risk, including a preoperative valgus alignment >10°, previous history of patellar instability, obesity (BMI >35), and generalized ligamentous laxity [4,15]. Recent in vivo evidence indicates that female patients demonstrate ~10° more lateral patellar tilt than males throughout flexion, potentially predisposing them to symptoms even when components are well aligned [5].  An elevated tibial joint line, referred to as pseudo-patella baja, is most commonly caused by under-resection of the tibia during attempts to optimise flexion gaps. This iatrogenic alteration has been associated with a mean proximal shift of approximately 4.2 mm and should be recognised as a significant risk factor for postoperative maltracking [5].

Diagnostic evaluation

Clinical Assessment

Common symptoms include anterior knee pain, particularly on stair descent (92% sensitivity), and mechanical symptoms like giving way or snapping [4]. The timing and pattern of pain onset often provide clues to its underlying cause. Sudden pain following a previously asymptomatic period typically suggests component failure or extensor mechanism disruption, whereas persistent pain since the time of surgery is more commonly associated with technical issues during the initial procedure [4]. On examination, a lateral patellar glide of >50% of the patella width is 87% specific for instability. A positive J-sign during active extension suggests lateral subluxation. An increased Q angle (>20° in females, >15° in males) is a red flag for lateral maltracking [16]. Retrospective analyses have shown that anterior knee pain in the setting of TKA, when combined with a positive J-sign and high Q angle, is predictive of surgical intervention in over 60% of cases [1].

Imaging Modalities

Radiographs remain essential. The Merchant view is critical, where a lateral patellar tilt >5° or lateral displacement >3 mm indicates instability [17]. The lateral view can reveal patella alta (Insall-Salvati ratio >1.2), predisposing to instability [18]. Weight-bearing anteroposterior and Rosenberg views can be useful adjuncts for assessing component loosening or joint line elevation, which indirectly suggest maltracking mechanisms [2].

Computed tomography remains the gold standard for evaluating component rotation, particularly using the Berger protocol [7]. Femoral rotation is measured as the angle between the surgical epicondylar axis and the posterior condylar line. Tibial rotation is assessed by measuring the angle formed between the tibial tubercle and the posterior margin of the tibial implant. A malrotation >5° in either component often necessitates revision [19]. When maltracking is suspected intraoperatively, CAS platforms that map the six degrees of patellar motion can distinguish rotational malposition from soft‑tissue tethering and quantify joint line elevation in real time, guiding immediate correction [5].  Intraoperative mapping with CAS has been shown to detect unanticipated joint line alterations and femoral sizing mismatches in up to 22% of complex primary cases [4].

Dynamic MRI is increasingly being used to assess real-time tracking and soft-tissue involvement, but remains limited by availability and cost.

Management strategies

Non-surgical Management

Conservative options are typically ineffective. Bracing and quadriceps strengthening may offer temporary relief in mild cases without component malposition, but success rates are below 15% [1]. A trial of physiotherapy targeting the VMO and biofeedback training may be justified in low-demand or non-operative candidates, but recurrence is common and the long-term benefit minimal [1]. Lateral patellar bracing and taping strategies can temporarily reduce lateral tilt but do not alter the underlying biomechanics or prevent progression in symptomatic patients [2].

Surgical Interventions

Component revision is mandatory when CT confirms malrotation >5° [20]. Complete revision is superior; partial revisions (replacing only the femoral or tibial component) fail in up to 70% of cases [21]. Five-year success rates approach 89% when component malrotation is corrected [22].

Soft-tissue procedures include lateral retinacular release, which is effective for isolated lateral tethering, particularly when the superior lateral geniculate artery is preserved to reduce avascular necrosis risk [23]. Alone, it fails in 60% of malrotated TKAs [24]. MPFL reconstruction, used for isolated medial insufficiency, typically involves a semitendinosus graft from the medial patella to the adductor tubercle [25]. Proximal realignment (the Insall technique) involves VMO advancement and medial capsular plication, with success rates around 85% in mild cases [26]. Distal realignment procedures, including the Elmslie-Trillat (medialization of the tibial tubercle) and the Fulkerson osteotomy (anteromedialization), are indicated when the Q angle exceeds 25°. Risks include nonunion (8%) and patellar tendon rupture (5%) [27]. When performed concurrently with component revision, distal realignment may significantly improve patellar engagement in deep flexion and reduce postoperative crepitus (Table 1) [2].

**Table 1:** Outcomes of surgical techniques by etiology MPFL: medial patellofemoral ligament

Etiology	Optimal procedure	Success rate
Femoral malrotation	Component revision	92%
Lateral soft-tissue tightness	Lateral release	75%
MPFL insufficiency	MPFL reconstruction	95%
Combined instability	Revision + realignment	85%

Salvage options include patellofemoral arthroplasty in cases of chronic dislocation or arthritis, and trochleoplasty for severe trochlear dysplasia [28].

A 2023 systematic review encompassing 21 clinical series reported that although component revision or soft‑tissue realignment restores anatomic tracking in most cases, 11%-25% of patients continue to report pain or dissatisfaction, underscoring the need for prevention at the index TKA [1].

Prevention strategies

Surgical Technique Refinements

For femoral component alignment, reliance on the epicondylar axis is key. Whiteside’s line serves as a secondary reference for femoral rotation, whereas posterior condylar referencing is considered unreliable in valgus knees [29]. In valgus knees, 3°-5° of external rotation is preferred [30]. The tibial component should be aligned with the medial third of the tibial tubercle, avoiding posterior slopes >7° [2]. During patellar resurfacing, 12-15 mm of bone stock should be maintained, and the patellar component should be medialized to reduce lateral release needs [9].

Intraoperative Assessment

Tourniquet deflation allows for more accurate tracking assessment, as tourniquets falsely simulate maltracking in nearly 48% of cases [31]. The no-thumb test helps assess patellar engagement without lateral support. A subvastus approach shows lower lateral release rates than medial parapatellar (23% vs. 52%) [32]. Surgeons may use CAS to visualise the distance between the planned femoral groove and the native patellar trajectory; adjusting femoral rotation or downsizing an oversized component based on this feedback has been shown experimentally to normalise tilt without recourse to lateral release [5]. The intraoperative use of computer navigation not only identifies rotational mismatch but also visualizes dynamic tracking, allowing immediate adjustment before cementation and improving first-time accuracy [1,4].

Implant Selection

The use of asymmetrical trochlear designs and appropriately sized femoral components is essential for optimizing patellar tracking. Modern anatomic implants, such as those with a J-curve design, have been shown to reduce maltracking complications by better accommodating native patellofemoral kinematics [33]. Avoidance of symmetrical, shallow trochlear implants in high-risk patients, particularly women with preoperative valgus, may be key in reducing the burden of postoperative instability [2].

Emerging Technologies

Computer-assisted surgery has been shown to reduce femoral rotational outliers from 28% to 9% [34]. While patient-specific instrumentation may offer similar theoretical benefits, long-term outcome data remain inconclusive [35]. Emerging navigation modules specifically designed to assess patellofemoral tracking intraoperatively show promise in further reducing maltracking-related errors, though larger clinical cohort studies are required to confirm this [5]. These tools may prove especially useful in complex primary or revision cases, where visualizing the patellar path dynamically can help avoid costly reoperation and improve component congruency from the outset [1,4].

## Conclusions

Patellar instability following TKA is a multifactorial complication that demands careful attention to both surgical technique and patient-specific variables. Accurate rotational alignment of femoral and tibial components remains critical, as does the restoration of soft-tissue balance and proper joint line height. Implant selection plays a significant role, with modern asymmetric trochlear designs offering improved patellar tracking. Computer-assisted technologies provide valuable intraoperative feedback, enabling real-time correction of malalignment and reducing the incidence of postoperative maltracking. Ultimately, a preventative approach, grounded in meticulous preoperative planning, intraoperative assessment, and individualized management, is essential to optimizing long-term function and minimizing the risk of revision in patients undergoing TKA.
